# A non-linear mixed effect model for innate immune response: *In vivo* kinetics of endotoxin and its induction of the cytokines tumor necrosis factor alpha and interleukin-6

**DOI:** 10.1371/journal.pone.0211981

**Published:** 2019-02-21

**Authors:** Anders Thorsted, Salim Bouchene, Eva Tano, Markus Castegren, Miklós Lipcsey, Jan Sjölin, Mats O. Karlsson, Lena E. Friberg, Elisabet I. Nielsen

**Affiliations:** 1 Pharmacometrics Research Group, Department of Pharmaceutical Biosciences, Uppsala University, Uppsala, Sweden; 2 Section of Clinical Microbiology and Infectious Medicine, Department of Medical Sciences, Uppsala University Hospital, Uppsala, Sweden; 3 Section of Infectious Diseases, Department of Medical Sciences, Uppsala University Hospital, Uppsala, Sweden; 4 Division of Perioperative Medicine and Intensive Care, Karolinska University Hospital, Stockholm, Sweden; 5 Department of Clinical Science, Intervention and Technology, Karolinska Institute, Stockholm, Sweden; 6 Hedenstierna Laboratory, Section of Anesthesiology and Intensive Care, Department of Surgical Sciences, Uppsala University Hospital, Uppsala, Sweden; University of Kansas Medical Center, UNITED STATES

## Abstract

Endotoxin, a component of the outer membrane of Gram-negative bacteria, has been extensively studied as a stimulator of the innate immune response. However, the temporal aspects and exposure-response relationship of endotoxin and resulting cytokine induction and tolerance development is less well defined. The aim of this work was to establish an *in silico* model that simultaneously captures and connects the *in vivo* time-courses of endotoxin, tumor necrosis factor alpha (TNF-α), interleukin-6 (IL-6), and associated tolerance development. Data from six studies of porcine endotoxemia in anesthetized piglets (n = 116) were combined and used in the analysis, with purified endotoxin (*Escherichia coli* O111:B4) being infused intravenously for 1–30 h in rates of 0.063–16.0 μg/kg/h across studies. All data were modelled simultaneously by means of importance sampling in the non-linear mixed effects modelling software NONMEM. The infused endotoxin followed one-compartment disposition and non-linear elimination, and stimulated the production of TNF-α to describe the rapid increase in plasma concentration. Tolerance development, observed as declining TNF-α concentration with continued infusion of endotoxin, was also driven by endotoxin as a concentration-dependent increase in the potency parameter related to TNF-α production (*EC*_*50*_). Production of IL-6 was stimulated by both endotoxin and TNF-α, and four consecutive transit compartments described delayed increase in plasma IL-6. A model which simultaneously account for the time-courses of endotoxin and two immune response markers, the cytokines TNF-α and IL-6, as well as the development of endotoxin tolerance, was successfully established. This model-based approach is unique in its description of the time-courses and their interrelation and may be applied within research on immune response to bacterial endotoxin, or in pre-clinical pharmaceutical research when dealing with study design or translational aspects.

## Introduction

Bacterial infection will invoke the host immune system and elicit a response of varying magnitude and duration depending on factors relating to the host and/or the invading pathogen. The prototypical pathogen-associated molecular pattern (PAMP) from Gram-negative bacteria is endotoxin (ETX), also called lipopolysaccharide, located in the outer membrane [1, 2]. Though ETX exhibits no toxicity *per se*, recognition by the immune system lead to a response aimed at clearing the pathogen to protect the host. However, an aggravated response can lead to tissue and organ injury, a clinical condition known as sepsis, defined as life-threatening organ dysfunction caused by a dysregulated host response to infection [3]. Sepsis is associated with high mortality rates and high per-patient costs of treatment in the intensive care unit and constitute a serious health care problem [4, 5].

The recognition of ETX through toll-like receptor 4 (TLR-4) on immune cells (e.g. tissue resident macrophages) will lead to a predominantly pro-inflammatory innate immune response involving the release of a plethora of cytokines in addition to activation and tissue infiltration of white blood cells [6–8]. Cytokines are endogenous mediators involved in a complex network of secondary reactions and cell-signalling, such as stimulation of acute phase protein secretion by the liver or activation of lymphocytes and thrombocytes [9]. Two key cytokines of innate immunity are tumor necrosis factor alpha (TNF-α) and interleukin-6 (IL-6) whose plasma concentration quickly increase over baseline values upon exposure to ETX, making them interesting biomarkers for the extent of early immune activation in a research setting [10].

While the *in vitro* and *in vivo* immune response to ETX is thoroughly investigated, a knowledge gap exists concerning the time-courses in the interplay between bacteria (or bacterial products) and host-response. Mathematical modelling is a key tool in the understanding of time-courses, and multiple models describing immune response to different infections are available. However, many of these are higly complex (alleviated by fixing certain parameters to literature values) [11, 12], discrete in nature and not able to describe time-courses [13], or not generalized for a population [14]. As alternative, a non-linear mixed effects modelling approach offers a simultaneous description of the typical tendency of data as well as consideration of the individual variability in a population, based on a set of ordinary differential equations. As such, data from multiple studies and individuals can be combined in the same model, and simulations of the system and its variability can be carried out according to different designs [15].

A wide range of pharmacokinetic-pharmacodynamic (PKPD) models have been developed to describe and understand the effects of antibiotics on bacteria [16]. These models are accompanied by a comprehensive collection of population PK models describing the time-course of antibiotics in different patient populations. However, the impact of the host immune system on the clearance of an infection, and the impact of the immune system on the physiological functioning of the host is usually disregarded. Development of models that describes the temporal aspects of immune activation is needed prior to a simultaneous integration of antibiotic and immune effects on a given pathogen, something that would be of value when selecting an appropriate antibiotic treatment regimen for optimizing therapy in patients [17].

In this work, we developed a mathematical model that simultaneously account for and connect the *in vivo* time-courses and interrelation of ETX, TNF-α and IL-6, and associated tolerance development, based on data from a large cohort of endotoxemic piglets.

## Results

**Fig 1:**
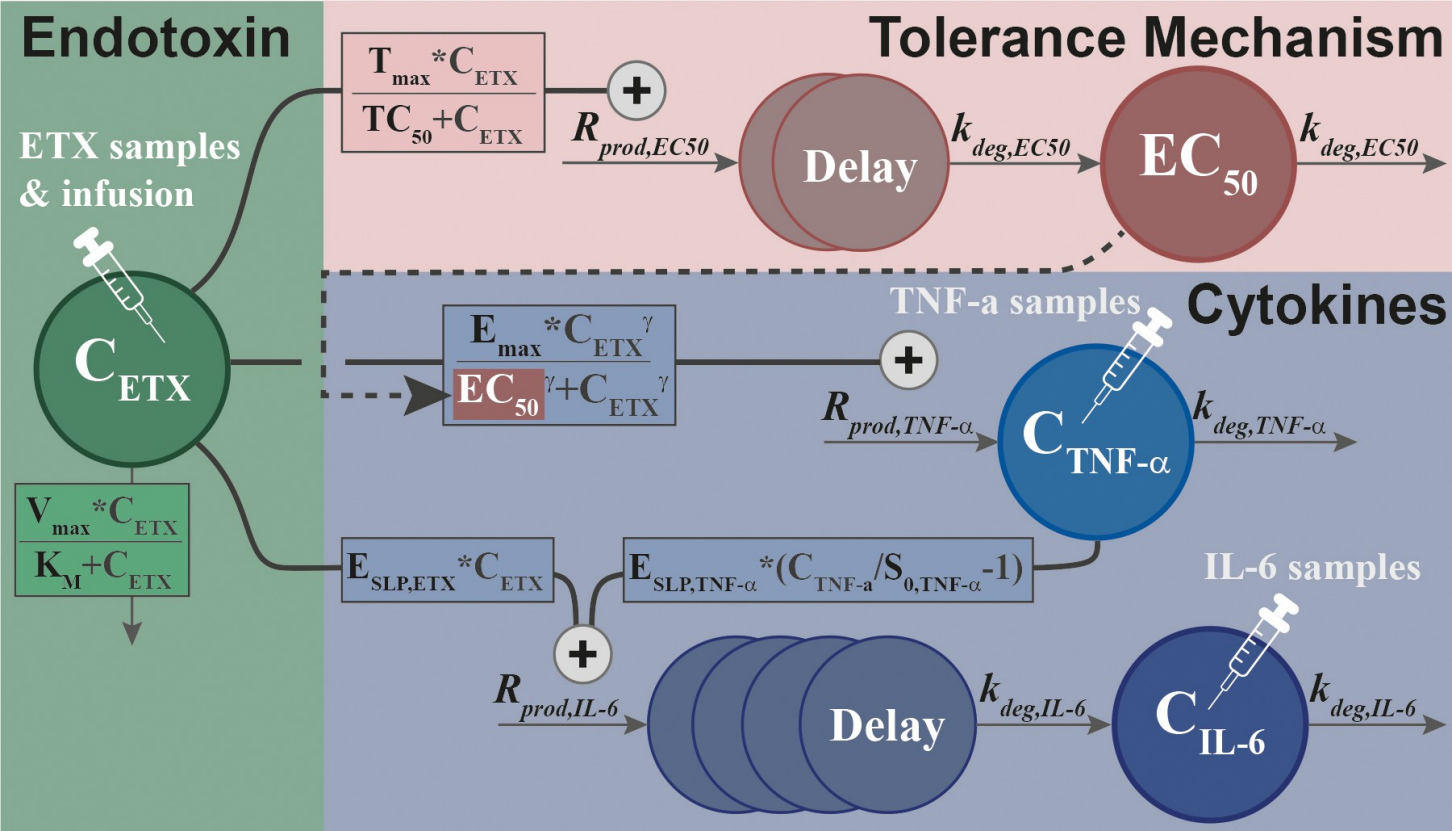
Schematic of the final model structure, linking the endotoxin concentration-time course (*C*_*ETX*_) to induction of tumor necrosis factor alpha (*C*_*TNF-α*_) through a sigmoidal Emax relationship, and to development of tolerance (*EC*_*50*_) through an Emax relationship. Both time-courses stimulate the induction of interleukin-6 (*C*_*IL-6*_) with linear relationships to endotoxin and tumor necrosis factor alpha. For parameter description and estimates, see **Table 2**.

The studies presented in **Table 1** formed the basis for the development of our integrated model (see respective reference for a thorough description), with one study (**G**) held out and used for external evaluation as it was only made available after the modelling work had been initiated. The dataset consisted of 116 individuals and 2391 observations (278 ETX, 1068 TNF-α, and 1045 IL-6) and led to the final model structure presented in **Fig 1**, with maximum likelihood estimates and variances as presented in **Table 2** (see **Methods**). The final dataset used during model development, as well as code describing the final model structure is included as supplementary information (**S1 Table** and **S1 Text**). Each sub-model is explained in detail in the following sections. In summary, ETX kinetics followed one-compartment disposition and saturable elimination, and the concentration-time course stimulated the production rate of TNF-α through a sigmoidal E_max_ relationship. Simultaneously, the ETX concentration-time course was responsible for a delayed increase in the potency parameter of the ETX-TNF-α relationship, thereby introducing tolerance of varying magnitude dependent on prior ETX exposure. The concentration-time courses of ETX and TNF-α simultaneously increased the production rate of IL-6, with appearance in plasma delayed through a chain of transit compartments.

**Table 1:** Overview of the studies used for model building (A-F) and external evaluation (G), including a short description of the design, number of animals, study length, infusion rates, assays and the amount of data points available for each modelled outcome.

Study	Short Description of the Design	N	Study Length	ETX Batch (EU/μg)	Infusion Rates (μg/kg/h)	Data	Samples (BLOQ)	Assay ^*a*^	(LOQ)	Reference
**A**	Twenty-four hour intravenous infusion of ETX at low rates, followed by six hour intravenous infusion of ETX at much higher rates to understand the development of ETX tolerance, including a control group with intravenous infusion of saline.	27	30 h	1000	Pre-exposure: 0; 0.063; 0.250	TNF-α	439 (0)	DuoSet	(NA)	[18]
						IL-6	422 (0)	DuoSet	(NA)	
					ETX-challenge: 0; 1.0; 4.0					
**B**	Twenty-four hour intravenous infusion of ETX at low rates with *ex vivo* stimulation to assess the development of ETX tolerance, including a control group with intravenous infusion of saline.	21	24 h	1000	0; 0.063; 0.250	TNF-α	186 (0)	DuoSet	(NA)	[19]
						IL-6	176 (0)	DuoSet	(NA)	
**C**	Six hour intravenous infusion of ETX to examine the dose(-exposure)-response relationship of ETX, including a control group with intravenous infusion of saline.	20	6 h	3000	0; 0.063; 0.250; 1.0; 4.0; 8.0; 16	ETX	75 (0)	Endochrome	(NA)	[20]
						TNF-α	132 (77)	BioSource	(10)	
						IL-6	132 (34)	Quantikine	(10)	
**D**	Three hour intravenous infusion of ETX, with accelerating or decelerating rates for each hour, in order to examine the impact of infusion rate on immune response and ETX tolerance.	18	6 h	3000	Accelerating: 0.063; 1.0; 4.0	TNF-α	117 (0)	BioSource	(NA)	[21]
						IL-6	117 (22)	Quantikine	(10)	
					Decelerating: 4.0; 1.0; 0.063					
**E**	One, two or three hour intravenous infusion of ETX at two different infusion rates, to mimic removal of ETX, including a control group with infusion of saline.	26	6 h	1000	0; 0.063; 4.0	ETX	178 (0)	Endochrome	(NA)	[22]
						TNF-α	175 (79)	DuoSet	(60)	
						IL-6	178 (41)	DuoSet	(60)	
**F**	Six hour intravenous infusion of ETX, starting at a higher infusion rate during the first thirty minutes.	4	6 h	3000	1.0; 4.0	ETX	28 (0)	Coatest	(NA)	[23]
						TNF-α	28 (13)	BioSource	(10)	
						IL-6	28 (4)	Quantikine	(10)	
**G**	Six hour intravenous infusion of ETX at a constant rate	6	6h	3000	0.5	TNF-α	42 (10)	DuoSet	(NA)	[24]
						IL-6	42 (0)	DuoSet	(200)	

Abbreviations: BLOQ: below limit of quantification; ETX: endotoxin; EU: endotoxin units; IL-6: interleukin-6; LOQ: limit of quantification; N: number; NA: not applicable; TNF-α: tumor necrosis factor alpha

^*a*^ Duoset: D686 (TNF-α) and DY690 (IL-6), R&D Systems, Minneapolis, MN, USA. Endochrome: Endochrome-K, Charles River Endosafe, Charleston, SC, USA. BioSource: KSC3011/KSC3012, Biosource International, Nivelles, Belgium. Quantikine: P6000, R&D Systems, Minneapolis, MN, USA. Coatest: Coatest Plasma Chromo-LAL, Charles River Endosafe, Charleston, SC, USA.

**Table 2:** Overview of parameters in the final model, with estimated value, uncertainty, inter-individual variability, and shrinkage.

Parameters	(unit)	Parameter Description	Estimates (RSE%)		Variability in CV% (RSE%)^*c*^ [SHR%]		
V_max_	(EU/h)	Maximum ETX elimination capacity	442000	(1)	-		
K_M_	(EU/L)	Concentration of ETX for half of V_max_	12600	(17)	120	(21)	[13]
V_c_	(L)	Volume of distribution for ETX	36.1	(20)	75.3	(25)	[24]
BASE_ETX_	(EU/L)	Baseline ETX (Endochrome, study C+E)^*b*^	155	(20)	96.3	(21)	[18]
		Baseline ETX (Coatest, study F)^*b*^	1810	(38)			
MIX ^*a*^	(-)	Proportion of individuals with contamination	0.399	(20)	-		
Prop_cont_	(-)	Proportionality: *BASE*_*ETX*_ to contamination	132	(15)	-		
MTT_TNF-α_	(h)	Mean transit time for TNF-α in plasma	1.04	(13)	81.5	(28)	[14]
S_0,TNF-α_	(ng/L)	Baseline TNF-α (DuoSet, study A+B)^*b*^	281	(9)	54.1	(23)	[13]
		Baseline TNF-α (DuoSet, study E)^*b*^	25.4	(12)			
		Baseline TNF-α (BioSource, study C+F)^*b*^	3.06	(29)			
		Baseline TNF-α (BioSource, study D)^*b*^	68.2	(15)			
E_max_	(-)	Maximum increase of TNF-α production rate	2540	(9)	-		
EC_0_	(EU/L)	Concentration of ETX at half of E_max,TNF-α_	286	(5)	-		
γ	(-)	Sigmoidicity coefficient for E_max_ relationship	2.10	(26)	-		
MTT_EC50_	(h)	Mean transit time for EC50_EC50,TNF-α_	6.33	(5)	-		
T_max_	(-)	Maximum increase of EC_0,TNF-α_	45100	(2)	-		
TC_50_	(EU/L)	Concentration of ETX at half of T_max_	29300	(3)	-		
MTT_IL-6_	(h)	Mean transit time for IL-6 in plasma	1.45	(11)	47.4	(41)	[21]
S_0,IL-6_	(ng/L)	Baseline IL-6 (DuoSet, study A+B+E)^*b*^	49.2	(13)	79.9	(10)	[6]
		Baseline IL-6 (BioSource, study C+D+F)^*b*^	8.19	(10)			
E_SLP,TNF-α_	(-)	Slope for TNF-α increase of R_prod,IL-6_	0.961	(9)	-		
E_SLP,ETX_	(-)	Slope for ETX increase of R_prod,IL-6_	0.00937	(28)	-		
σ _ETX_	(%)	Proportional error for ETX	32.1	(16)			[23]
σ _TNF-α_	(%)	Proportional error for TNF-α	48.5	(8)			[11]
σ _IL-6_	(%)	Proportional error for IL-6	50.5	(8)			[11]

Abbreviations: CV%: coefficient of variation; ETX: endotoxin; EU: endotoxin units; IL-6: interleukin-6; RSE%: relative standard error in percent; SHR%: shrinkage in percent; TNF-α: tumor necrosis factor alpha

^*a*^ parameter was fixed in final estimation, and the RSE% is from the final run of the ETX modelling

^*b*^ The letters (A-F) refer to the study as designated in **Table 1**.

^*c*^ Reported on the approximate standard deviation scale (standard error/variance estimate)/2.

### Infused endotoxin

The infused ETX was distributed according to a one-compartmental model, with elimination described as a non-linear relationship to concentration, and a constant assay-dependent baseline (*BASE*_*ETX*_) added to the ETX predictions. Addition of a peripheral compartment was not significant according to the statistical measure of model fit (change in objective function value, *ΔOFV* = -4.29 *vs*. one-compartment), but the non-linear elimination resulted in an improved fit (*ΔOFV* = -12.9 *vs*. linear). Terms describing inter-individual variability (IIV) was supported for the parameters *K*_*M*_, *V*_*C*_ and *BASE*_*ETX*_, with a negative correlation between *K*_*M*_ and *V*_*C*_ (-0.42).

**Fig 2:**
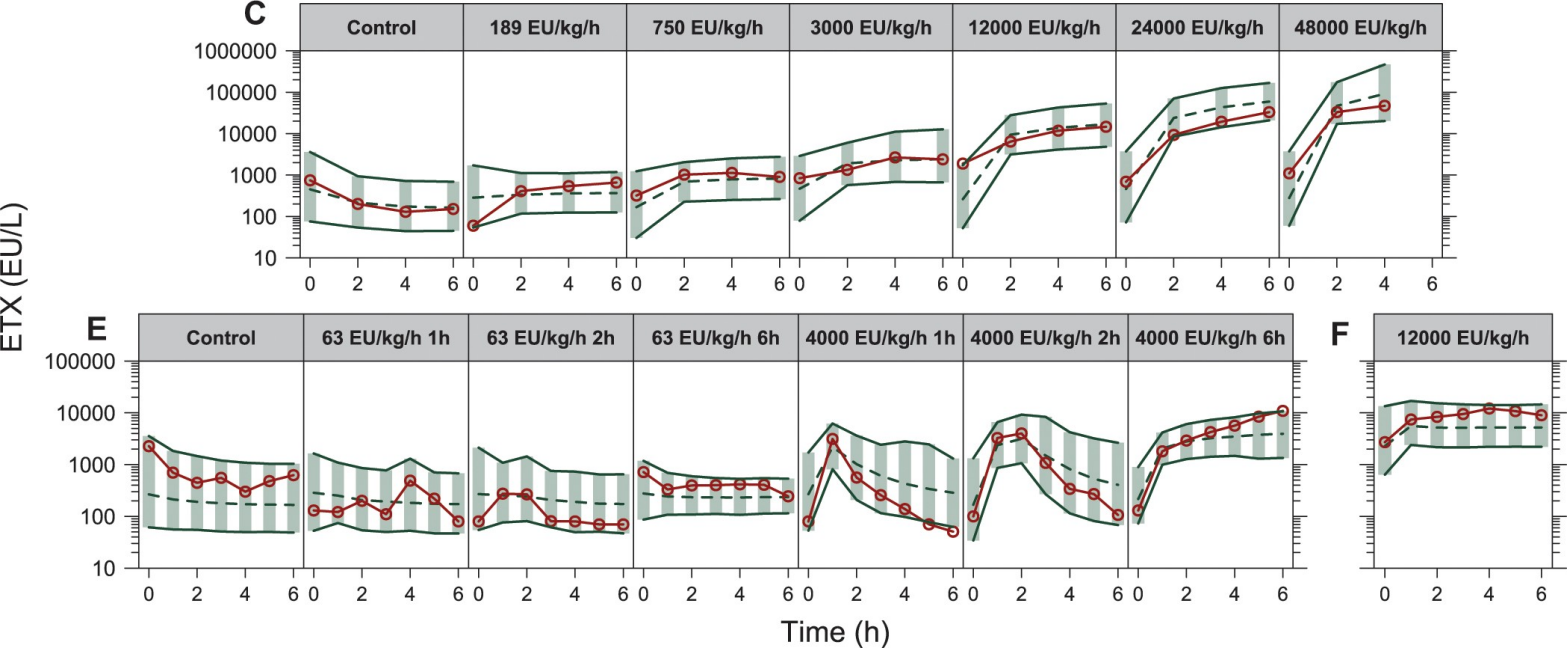
Visual predictive checks for endotoxin based on the final model, stratified by study and infusion rate or design (studies C, E & F in Table 1), with the endotoxin infusion rate indicated for each panel. The red solid lines and circles correspond to the median observed concentration, while the dashed line correspond to the median simulated concentration. The shaded area between the two solid lines correspond to the 95% confidence interval for the median simulation.

An indication of contamination was observed in early samples for a proportion of the piglets, with ETX concentrations higher than the modelled constant ETX baseline (most obvious in control and low-dose groups shown in **Fig 2**). This was handled by implementing a mixture model describing two sub-populations. The central compartment for sub-population one was initialized to zero, whereas it was initialized to a quantity directly proportional to *BASE*_*ETX*,*i*_ for sub-population two, as described by:

$$A_{ETX,i,t=0}={BASE}_{ETX,i}*{Prop}_{cont}$$ (1)

This initial amount was allowed to decay over time together with the infused ETX, and significantly improved the fit (*ΔOFV* = -80.8), the residual and simulation based diagnostics, and decreased the residual unexplained variability (RUV).

### Host response: TNF-α and IL-6

The relationship between ETX exposure, a potent TLR-4 agonist, and host response measured by TNF-α was initially established by letting the infused ETX stimulate the production rate, similar to the turnover model shown in Eq 1. As control groups showed no cytokine response, neither the constant baseline ETX level nor the contamination, described above, contributed to the stimulation. The best description of tolerance was obtained when the concentration time-course of ETX–simultaneously to driving TNF-α response–modified the value of *EC*_*50*_, i.e., *EC*_*50*_ was a function of ETX exposure. This was done through an E_max_ relationship (*ΔOFV* = -122.7 *vs*. linear), with a delay in the increase of *EC*_*50*_ described by two transit compartments (*ΔOFV* = -894.5 *vs*. no transits). With this tolerance model, a sigmoidal E_max_ relationship between ETX and TNF-α was significant (*ΔOFV* = -250.1 *vs*. standard E_max_). A large variation was observed in the baseline TNF-α (*S*_*0*,*TNF-a*_) among the included studies, and different baselines were tested on a per-study basis. This resulted in four different typical baselines (*ΔOFV* = -120.5 *vs*. one shared), varying from 3.06 to 281 ng/L, with higher values for the DuoSet assay. However, the relative increase in TNF-α at a certain exposure of ETX did not differ across the studies, as there was no significant differences in *E*_*max*_.

The model was extended to IL-6 by letting TNF-α stimulate the production of IL-6 through a linear relationship. As different baseline TNF-α (*S*_*0*,*TNF-a*_) were used among the studies the relative increase was used to drive the effect, as in:

$${EFF}_{TNF-\alpha }=E_{SLP,TNF-\alpha }*(\frac{C_{TNF-\alpha }}{S_{0,TNF-\alpha }}-1)$$ (2)

where *E*_*SLP*,*TNF-α*_ describes the proportionality between relative increase in TNF-α and stimulation of IL-6 production. To describe the delayed increase in plasma IL-6, four transit compartments were added to the model (*ΔOFV* = -255.7 *vs*. no transits). In addition, an effect of the ETX time-course (linear relationship) on the production of IL-6 was found significant (*ΔOFV* = -268). No improvements could be identified with more complex effect models (E_max_, sigmoidal E_max_) for either TNF-α or ETX. As for TNF-α, differences in baseline (*S*_*0*,*IL-6*_) was significant (*ΔOFV* = -83.6), with one estimated baseline per assay (8.19 and 49.2 ng/L).

Inclusion of IIV terms were significant for baselines (*S*_*0*,*TNF-a*_ and *S*_*0*,*IL-6*_) and the first-order rate constants describing cytokine degradation (*k*_*deg*,*TNF-α*_ and *k*_*deg*,*IL-6*_ in **Fig 1**). Correlation in the individual empirical Bayes’ estimates (EBEs) were also assessed (e.g. high *S*_*0*,*TNF-a*_ could correspond to high *S*_*0*,*IL-6*_), but no such relations were identified. Lastly, for all three outcomes the initial additive model on log-transformed data were adequate for description of RUV.

**Fig 3:**
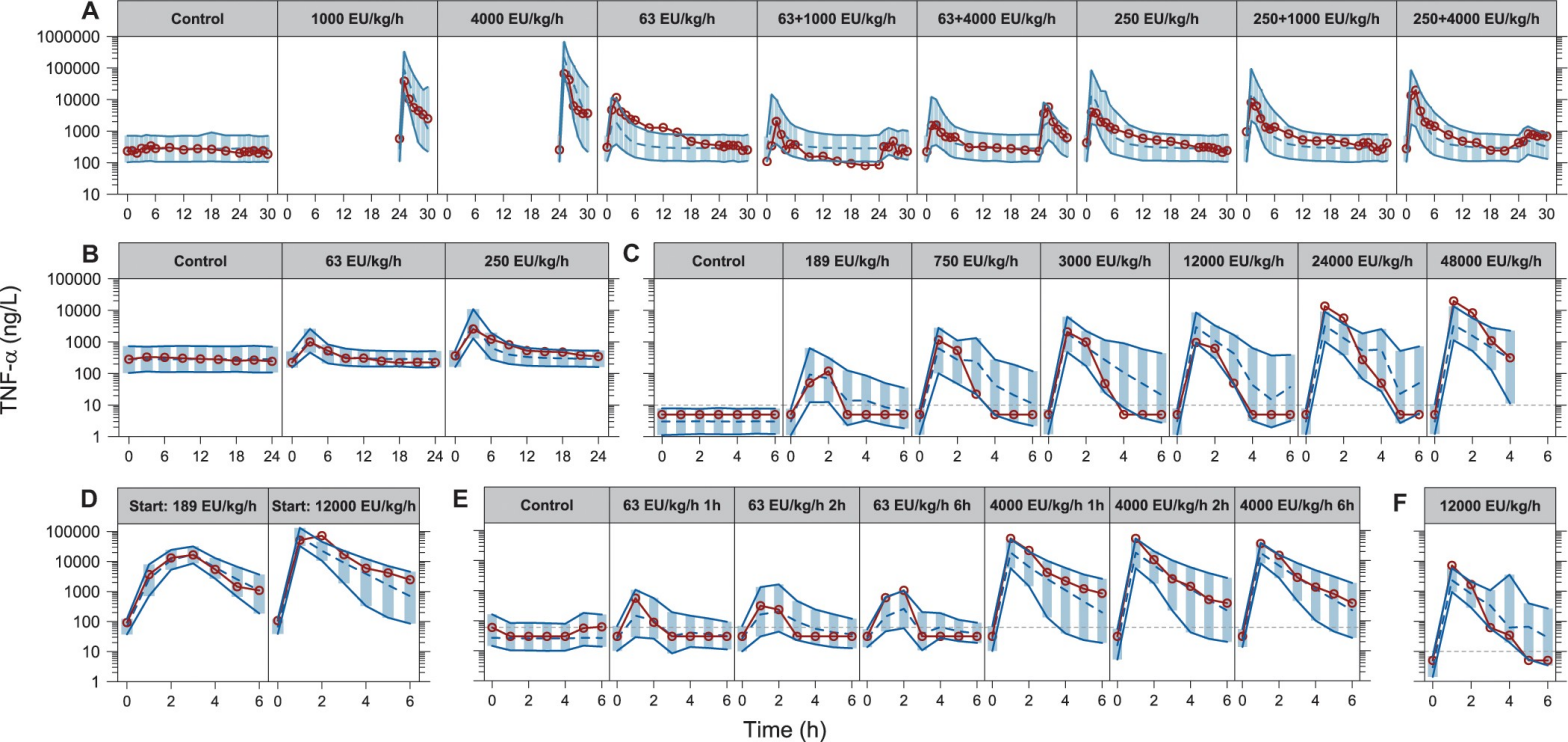
Visual predictive checks for tumor necrosis factor alpha based on the final model, stratified by study and infusion rate or design (studies A-F in Table 1), with the endotoxin infusion rate indicated for each panel. The red solid lines and circles correspond to the median observed concentration, while the dashed line correspond to the median simulated concentration. The shaded area between the two solid lines correspond to the 95% confidence interval for the median simulation. The dashed horizontal lines corresponds to the assay lower limit of quantification, with observations below set to half of this limit for illustration.

**Fig 4:**
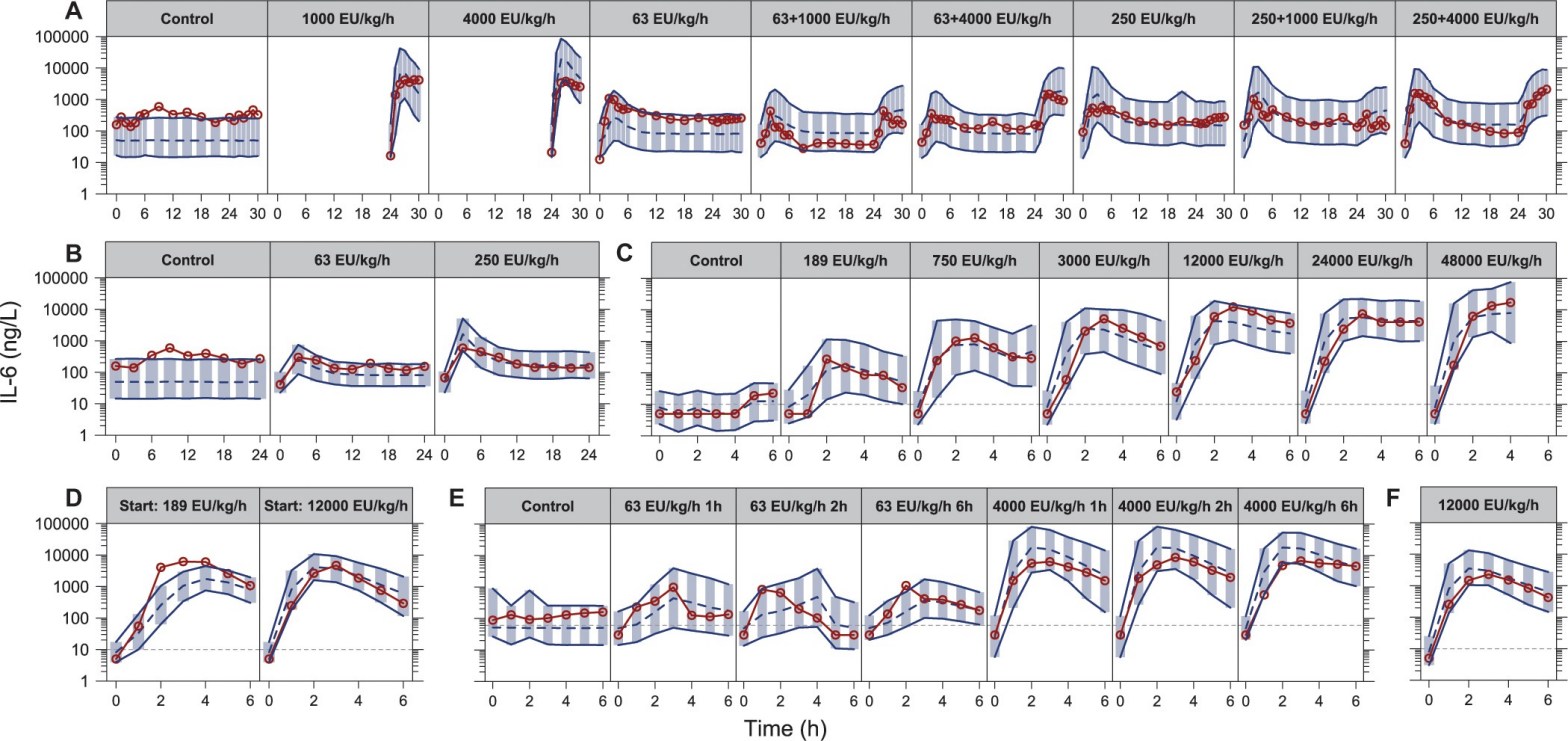
Visual predictive checks for interleukin-6 based on the final model, stratified by study and infusion rate or design (studies A-F in Table 1), with the endotoxin infusion rate indicated for each panel. The red solid lines and circles correspond to the median observed concentration, while the dashed line correspond to the median simulated concentration. The shaded area between the two solid lines correspond to the 95% confidence interval for the median simulation. The dashed horizontal lines corresponds to the assay lower limit of quantification, with observations below set to half of this limit for illustration.

The final model was run with all structural parameters and variances unfixed, except the parameter describing the mixture proportion for ETX contamination. This parameter was fixed to the final estimate in the ETX sub-model (0.399, describing a proportion of individuals), because the inclusion of individuals without ETX observations led to instability in the estimation of this parameter. The final fit was evaluated using prediction corrected visual predictive checks (VPCs) stratified by study and infusion rate or design (shown in **Fig 2** for ETX, **Fig 3** for TNF-α and **Fig 4** for IL-6). Generally, all panels showed that the medians of the observations were well predicted by the model for the different infusion rates and designs. To assess if the included IIV terms were adequate, prediction corrected VPCs were stratified by study only to compare the observed and simulated outer percentiles, which also confirmed an acceptable description.

### External evaluation

**Fig 5:**
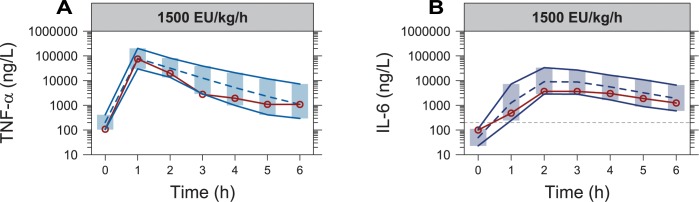
Visual predictive checks for observations of (**A**) tumor necrosis factor alpha and (**B**) interleukin-6 from an external dataset (study G in **Table 1**), with the endotoxin infusion rate indicated for each panel. The red solid lines and circles correspond to the median observed concentration in the external dataset, while the dashed line correspond to the median simulated concentration based on the final model. The shaded area between the two solid lines correspond to the 95% confidence interval for the median simulation. The dashed horizontal line in (**B**) corresponds to the assay lower limit of quantification (200 ng/L), with observations below set to half of this limit for illustration.

In the held-out data (study **G** in **Table 1**) both cytokines were assayed using DuoSet [24], though all IL-6 baseline values were set to 200 ng/L (employed limit of quantification (LOQ) in for this data). Because of the varying TNF-α baselines between studies using the DuoSet assay, and because all baseline measurements of IL-6 were below the LOQ, the median of the individual EBEs were used as the typical values when simulating from the final model (205 and 49.3 ng/L for TNF-α and IL-6, respectively). All other parameters and variances were set to the final values reported in **Table 1**. The VPC shown in **Fig 5** demonstrates a good agreement between the observed median cytokine time-course in the held-out data, and the median and its confidence interval based on simulations from the final model.

### Illustrating model properties

**Fig 6:**
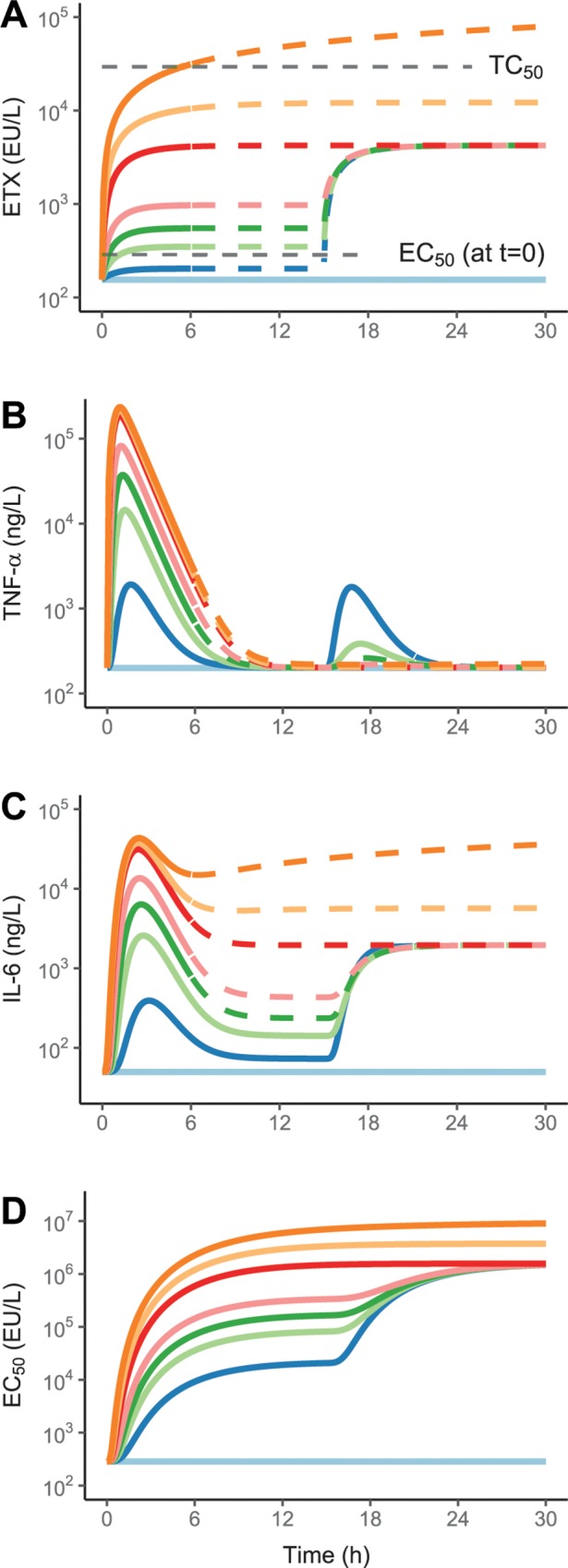
Final model predictions of the time-courses of endotoxin (**A**), tumor necrosis factor alpha (**B**), interleukin-6 (**C**), and the potency parameter *EC*_*50*_ (**D**). Different initial infusion rates of endotoxin, in EU/kg/h, were used: Control (designated with a light blue line), 63 (blue), 250 (light green), 500 (green), 1000 (pink), 4000 (red), 8000 (yellow), and 16000 (orange), changed to 4000 EU/kg/h after 15 h for the lower rates of 63, 250, 500, and 1000 EU/kg/h, to demonstrate tolerance aspects. The dashed part of the lines indicate extrapolation beyond the period of the observed data.

**Fig 7:**
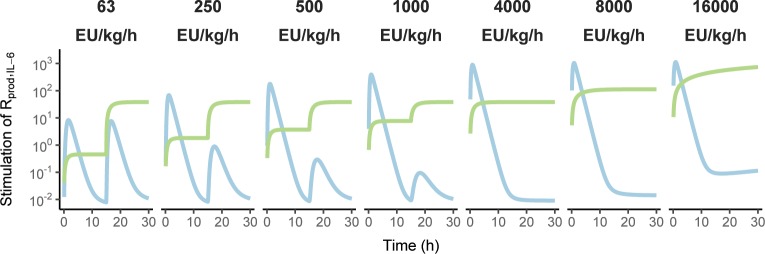
The respective contribution of endotoxin (light green line) and tumor necrosis factor alpha (light blue line) time-courses on the stimulation of interleukin-6 production. The initial infusion rate of endotoxin is indicated in each panel description, changed to 4000 EU/kg/h after 15 h for the lower rates of 63, 250, 500, and 1000 EU/kg/h to demonstrate tolerance aspects.

Predictions for a 30 h period were done for infusion rates similar to those in the available data (63–16000 EU/kg/h, assuming an ETX batch with a potency of 1000 EU/μg), with the lower rates changed to 4000 EU/kg/h after the first 15 h to illustrate tolerance aspects. The predictions of ETX, TNF-α and IL-6 as well as the dynamical change in *EC*_*50*_ are shown in **Fig 6**, with dashed lines indicating extrapolation outside the available data (primarily for high infusion rates). It is observed for model predictions of TNF-α, that initial infusion rates above 250 EU/kg/h limits the possibility to further elicit a response by increasing the infusion rate at a later time-point. Furthermore, it can be seen for model predictions of IL-6, that the highest infusion rates leads to consistently elevated IL-6 concentrations in the extrapolated parts, due to the constant ETX-associated stimulation becoming increasingly impactful as the infusion rate increases. This aspect is illustrated in **Fig 7** utilizing the same design, where the contribution of TNF-α to stimulation of IL-6 production is transitory and more-or-less negligible at 15 h for the highest infusion rates, whereas the stimulation by ETX is constant.

## Discussion

The presented work is, to our knowledge, the first that provides a non-linear mixed-effects model-based assessment of the ETX time-course and its effect on the production of two important cytokines in the innate immune response, TNF-α and IL-6. The developed model is based on a comprehensive dataset with a wide range of ETX infusion rates, administered over varying durations and under different designs. The studies were carried out in large mammals, expected to resemble humans with respect to physiology and immunological properties [25], which may be key for future extrapolation of the results.

The observed ETX time-course was adequately described (see **Fig 2**) using a one-compartment model with saturable elimination, in line with observed differences in half-life between two dose-groups in rhesus monkeys [26]. Further, radioactively labelled ETX are primarily found in plasma, liver, and spleen, the two latter being part of the reticuloendothelial system responsible for the (saturable) systemic elimination of ETX [27]. Two-compartmental kinetics has been reported [26, 28], but the combination of intravenous infusion, hourly sampling, and lack of elimination phase samples in our data, limited the determination of a more complex distribution model. The presented model was built on ETX concentration data following a wide range of infusion rates, unique and valuable in light of the challenges with ETX determination [29]. Important for this work, a model for the ETX concentration-time course is critical in terms of exposure-response where, under non-linear kinetics, change in exposure may not be proportional to the change in infusion rate. Nonetheless, the model would benefit from addition of ETX samples collected over longer durations than six hours and with more intensive sampling during the elimination phase.

The time-course of TNF-α displayed a rapid increase with peak concentration at the one-hour sample for most individuals, followed by a return towards baseline levels [30]. With a clear relationship between ETX infusion rate and peak TNF-α [20], the starting point was to let the ETX concentration-time course, a surrogate for TLR-4 activation [31], stimulate the TNF-α production rate. Even though this could describe the peaks, extension of the model was needed to handle the development of tolerance, implemented by letting the ETX concentration-time course describe a (delayed) increase in the potency parameter (*EC*_*50*_). The implementation was possible due to the study design with ETX infusions allowing for the separation of ETX and TNF-α time-courses. Alternative methods could have relied on anti-inflammatory mediators [32] to, for instance, negate the stimulation caused by ETX, but these were not available in the current dataset. In **Fig 3A**, the implementation of tolerance describes the difference in TNF-α response following a 4000 EU/kg/h ETX challenge, after two different pre-exposures of 63 and 250 EU/kg/h. A pronounced response to the challenge is observed following low pre-exposure, whereas a response is barely seen following the high pre-exposure, with both responses much lower than without any pre-exposure. The model was also able to describe differences observed following a decelerating or accelerating ETX infusion design (**Fig 3D**), and while there are signs of slight differences in TNF-α trajectory between studies, the model provides a good general description of TNF-α across the included data.

The model was extended to IL-6, whose peak plasma concentration is delayed compared to TNF-α [30]. This was handled by letting the time-course of TNF-α stimulate the production of IL-6, with addition of transit compartments to delay the increase in the IL-6 plasma concentration. This is appropriate, as it has been demonstrated that administration of TNF-α, independently of ETX, can increase IL-6 [33, 34], and as the tolerance already included for TNF-α will then be directly reflected in the time-course of IL-6. However, the model was improved when the time-course of ETX also contribued to the stimulation of IL-6 production, reflecting that the optimal description of immune system components is multifaceted with many interrelated stimulations and inhibitions. That both effects on IL-6 production follow a linear relationship could be because of difficulties in separating the effects under more complex models, because non-linearity is already captured in the established relation between ETX and TNF-α, or because the stimulation in the underlying data was not high enough to reach saturation. However, all fits presented in **Fig 4** show that the final model acceptably captures the observed IL-6 time-courses.

The ability of the model to predict the held out study, including an infusion rate of 1500 EU/kg/h that was not available among the studies used for model building, is shown in **Fig 5**. The good agreement between the observed data and model predictions demonstrate, that the model can be extended to predict piglet studies outside the modelled data, an aspect that adds certainty in prediction of new studies and generalisation of the model. The model structure suggested might also be useful in predicting studies in other species but would likely require information on cytokine baselines and any differences in susceptibility to ETX that would influence the exposure-response relationship established in piglets (likely *EC*_*50*_).

Improved understanding of model behaviour may be gained from the 30 h predictions shown in **Fig 6**, illustrating how the modelled outcomes respond across the available ETX infusion rates and how the value of *EC*_*50*_ changes dynamically as a function of ETX exposure. In addition, **Fig 7** shows the increasing contribution of ETX to IL-6 stimulation with higher infusion rates, whereas the contribution from TNF-α and ETX is approximately equal at the lower infusion rates, with TNF-α responsible for the peak. The high contribution from ETX at the higher infusion rates cannot be confirmed based on the current data, and as mortality occurred within the confines of a six-hour period [20], it is doubtful that such data could be obtained. As such, it is unclear if the high constant concentrations of IL-6 at infusion rates at or above 4000 EU/kg/h are realistic, or if it is only an extrapolation of the linear relationship between ETX and IL-6, valid for the first six hours and lowest infusion rates.

A previous analysis based on similar data linked measurements of cytokines and organ function based on literature and inferences from a principal component analysis, with adaption of values to data from individual pigs [14]. However, even though the pigs received similar treatment, large differences in parameters were required to describe differences in observations. This is in contrast to the model presented here, which simultaneously describes the general data tendency (the “typical” piglet) as well as each individual piglet profile (posthoc), through fixed effects and associated variances.

A recently published analysis [35] utilizing a modelling approach more similar to the one presented here, relied on a hypothetical unobserved compartment of anti-inflammatory cytokines to model inhibition of TNF-α and IL-1β release. This approach would work similarly to our dynamic change in *EC*_*50*_ for the acute tolerance, as both approaches ties cytokine inhibition directly to the pathogen burden. It is likely however, that some modifications would be needed to describe the lowered cytokine response upon a second ETX expsoure, though the most ideal scenario in both cases would be the sampling and availability of key anti-inflammatory cytokines or other measures of pro-inflammatory counter-response.

The presented work is the first step in a more comprehensive model-based framework of immune-system response to a bacterial marker, with potential extension to additional markers of inflammation, anti-inflammation or organ dysfunction. This could for instance include immune cells (e.g. neutrophils and lymphocytes) and circulatory or respiratory changes (e.g. mean pulmonary arterial pressure). The potential of coupling ETX to organ dysfunction is interesting, as the underlying animal model is expected to have similarities with humans, especially with regards to circulatory function [25]. A model-based framework could ultimately inform treatment decisions in the ICU where critical illness, such as sepsis, has a large impact on physiological processes such as fluid shifts, permeability, cardiac output or kidney function, underlying the PK–and thus PD–of therapeutic drugs [36, 37]. Further, while the PK behavior of antibiotics has been studied in both healthy volunteers and patients, the established differences has, to our knowledge, not been explained by directly linking changes in the underlying organ function to the changes in the PK of patients.

The work presented here can be applied to and aid in the development of new treatments within immunology, by informing early animal experiments (ETX dose and administration design, choice of sampling times, reducing the number of animals). The administration of purified *E*. *coli* ETX allows the TLR-4 induced activation of the immune response to be studied with a controlled exposure, which is not possible when using live bacteria. However, the established relation between ETX, TNF-α and IL-6 could be further extended by incorporating data on the release of ETX from live bacteria in the context of antibiotic administration [38, 39]. Further, data from the response following administration of living bacteria, either intravenously or as a localized infection, could be incorporated to model how such a cytokine response differs from the fixed TLR-4 response presented here [40, 41]. This could form the basis for predictive links between the porcine endotoxemia model and the ETX challenge model in healthy volunteers [30], where doses are limited and not close to establishing sepsis.

To conclude, the presented work established a non-linear mixed effects model for administered ETX and its induction of the two inflammatory cytokines TNF-α and IL-6. The model can be used as a basis for the understanding immune response to bacterial infection with different bacterial loads and for the simulation of new studies to answer design questions and/or inform pre-clinical studies.

## Materials and methods

### Ethics statement

This work relied on previously published data that was generated in studies involving live animals. Animals were handled according to guidelines of the Swedish National Board for Laboratory Animals and the European Convention on Animal Care, and study designs and procedures were approved by the Animal Ethics Committee of Uppsala, Sweden (Uppsala djurförsöksetiska nämnd, permit no. 215/5).

### Data

The model development was based on data from six pre-clinical *in vivo* studies carried out by the same research group and under similar conditions. The studies, including a seventh study used for external evaluation, are described shortly in **Table 1**, accompanied by information on the number of individuals, ETX infusion rates, number of samples, samples below the limit of quantification (BLOQ), assays and their LOQ, with additional information available in the respective publications. In total, the analysis was based on 281 ETX samples, 1077 TNF-α samples (169 BLOQ) and 1053 IL-6 samples (101 BLOQ), from 116 piglets. Prior to analysis, 27 of the observations (spread across individuals and outcomes) were excluded as outliers (obvious deviation from the individual concentration-time profiles).

#### Study design

All studies were performed in anaesthetized piglets with an age of approximately 12 weeks and a mean weight of approximately 27 kg. Anaesthesia and preparatory procedures were similar across the studies, with reference to respective publications for additional information given in **Table 1.**

The studies used *Escherichia coli*: 0111:B4 ETX (Sigma Chemical, St. Louis, MO) as an active agent, though the potency (i.e. endotoxin units (EU) per mass) of batches varied as shown in **Table 1**. The ETX was administered as continuous intravenous infusions at rates adjusted to the individual weight of the piglet, spanning from zero (saline) to 16 μg/kg/h, but referred to and handled in the model as EU/kg/h. Samples for ETX and cytokines were taken prior to ETX administration and then hourly to every third hour for the rest of the study duration. A range of other variables were measured, for example measurements related to organ dysfunction and physiological state, as well as immune cell-counts and haematology, but this analysis focused on the cytokines TNF-α and IL-6.

#### Bioanalytical assay

For ETX, analysis was performed using two different commercial chromogenic limulous amoebocyte lysate assays without a defined LOQ [42]. For the cytokines, three commercial sandwich enzyme-linked immunosorbent assay kits were used, with or without a defined LOQ (10 ng/L or 60 ng/L) dependent on the study (see **Table 1**).

### Software

The nonlinear mixed-effects modelling software NONMEM 7.4.2 (ICON Development Solutions, Hanover, MD, US) [43] was used for estimating model parameters, with Perl-speaks-NONMEM and Piraña used for facilitating model execution and handling the modelling workflow [44]. NONMEM models were executed on a Scientific Linux 6 computing cluster running Intel Xeon E5 nodes. R [45] was used for handling of raw data and analysis of NONMEM output with packages xpose4 [46], ggplot2 [47] and tidyr [48].

### Modelling process

The available data were modelled in a sequential order, initially aiming at describing the distribution and elimination of the infused ETX, its induction of TNF-α shown as a rapid increase in plasma concentration, and lastly extended to IL-6. With acceptable results for one outcome all parameters would be fixed in the following step, with the final run leaving all parameters unfixed for a simultaneous fit [49]. All data were log-transformed and estimated using importance sampling with interaction. As a large portion of the data, especially baseline cytokine samples, were BLOQ the likelihood based M3 method [50] was implemented, with the Laplacian method included for this extension.

Evaluation of model fits were based on likelihood-ratio tests of the OFV of two nested models, the precision of the parameter estimates (relative standard error, RSE%), and the adequacy of residual goodness-of-fit plots and appropriately stratified simulation-based VPCs [51]. IIV was included to produce lognormal distributed parameters, and an additive relation was used for RUV (approximately proportional with log-transformed data).

### Infused endotoxin

One- and two-compartment models were tested with either linear or non-linear elimination pathways. In addition, different approaches were taken to describe the baseline ETX level (or assay baseline) primarily informed by the samples from the control groups and the pre-dose measurements, as well as a general tendency of high ETX observations in some individuals before the infusion of ETX or saline was started (evident across dose groups).

### Host response: TNF-α and IL-6

A framework of turnover models were chosen to describe the change in cytokine concentration, as their parameterization provide parameters that are relatable in a biological context. Turnover models consist of a zero-order production rate (*R*_*prod*_), a first-order rate constant describing degradation (*k*_*deg*_), and a baseline (*S*_*0*_*)* given by the ratio *R*_*prod*_*/k*_*deg*_. The system can be disturbed, for instance by stimulating the production rate through an E_max_ relationship to the ETX concentration (*C*_*ETX*_), as described for TNF-α by:

$$\frac{dC_{TNF-\alpha }}{dt}=R_{prod,TNF-\alpha }*\left(1+\frac{E_{max}*C_{ETX}}{{EC}_{50}+C_{ETX}}\right)-k_{deg,TNF-\alpha }*C_{TNF-\alpha }$$ (3)

where *E*_*max*_ describes the maximum effect and *EC*_*50*_ is the potency (*C*_*ETX*_ for half maximum effect), with the baseline (*S*_*0*,*TNF-α*_) given by:

$$S_{0,TNF-\alpha }=\frac{R_{prod,TNF-\alpha }}{k_{deg,TNF-\alpha }}$$ (4)

Extensions of this system were explored, e.g. transit compartments were added to describe delays and functions describing development of tolerance were explored. Time-dependent changes in either *E*_*max*_ or *EC*_*50*_ were initially considered, but as reliance on time is not optimal for predictive capability or for capturing the tolerance dynamics of repeated ETX administration, models independent of time were desired as a final implementation [52].

### External evaluation

Data from a study that was not used in the model building process [24] was available to evaluate the final models’ ability to predict the observed TNF-α and IL-6 time-courses. The external evaluation was performed by comparing the median of the observations from this separate study, to simulations from the final model using the design of the external study. The cytokine baselines were derived from the median of the EBEs for those individuals analysed with the corresponding assay.

## Supporting information

S1 Table Raw endotoxin and cytokine dataset used in model development. Dataset used in development of the model, including information on each individuals administration of ETX and observed ETX, TNF-α and IL-6 concentrations at given times, plus any important information of the respective studies with regards to study design or similar.
(CSV)

S1 Text Final model file including differential equations. Model control file for the final model, showing input, compartments, parameters, initialization and differential equations, predictions of observations and observations below the limit of quantification, initial estimates for parameters and variances and estimation method.
(PDF)
