# Supplementary material for: A non-linear mixed effect model for innate immune response: In vivo kinetics of endotoxin and its induction of the cytokines tumor necrosis factor alpha and interleukin-6
Source: PLoS One. 2019 Feb 21;14(2):e0211981. doi: 10.1371/journal.pone.0211981 (PMC6383944; doi:10.1371/journal.pone.0211981)
Supplement: S1 Text — Model control file for the final model, showing input, compartments, parameters, initialization and differential equations, predictions of observations and observations below the limit of quantification, initial estimates for parameters and variances and estimation method. (PDF) [file pone.0211981.s002.pdf]

```
$PROBLEM    combined model
$INPUT      ID SID STUDY DGRP AMT1 AMT2=AMT RATE1 RATE2=RATE CMT EVID
            DVID TIME DV NMDV MDV OMIT LLOQ TYPE WT FLUID INFL INFR PEXP
$DATA       dataset.csv
            IGNORE=@
            IGNORE=(DVID.GE.3,OMIT.EQ.1)

$SUBROUTINE ADVAN6 TOL6
$MODEL      COMP=(ETX DEFDOSE DEFOBS) ; EU/L
            COMP=(DUMMY)
            COMP=(TNFA)               ; ng/L
            COMP=(1EC50)
            COMP=(2EC50)
            COMP=(3EC50)
            COMP=(IL6TR1)
            COMP=(IL6TR2)
            COMP=(IL6TR3)
            COMP=(IL6)
            COMP=(IL6TR4)

$MIX
NSPOP = 2
P(1) = 1 - THETA(7)
P(2) = THETA(7)

$PK
; --- ENDOTOXIN
MU_1 = LOG(THETA(1))
MU_2 = LOG(THETA(2))
MU_3 = LOG(THETA(3))

TVEBASE = THETA(4)
IF (STUDY == 6) TVEBASE = THETA(5)
MU_4 = LOG(TVEBASE)

MU_5 = LOG(THETA(6))

VMAX = EXP(MU_1 + ETA(1))
KM = EXP(MU_2 + ETA(2))
V1 = EXP(MU_3 + ETA(3))
EBASE = EXP(MU_4 + ETA(4))
PROP = EXP(MU_5 + ETA(5))

SOIL = 0
IF (MIXNUM == 2) SOIL = EBASE * PROP

; --- TNFA
MU_6 = LOG(THETA(8))

TVS0_TNF = THETA(9)
IF (STUDY == 3 .OR. STUDY == 6) TVS0_TNF = THETA(10)
IF (STUDY == 4) TVS0_TNF = THETA(11)
IF (STUDY == 5) TVS0_TNF = THETA(12)
MU_7 = LOG(TVS0_TNF)

MU_8 = LOG(THETA(13))
MU_9 = LOG(THETA(14))
MU_10 = LOG(THETA(15))
MU_11 = LOG(THETA(16))
MU_12 = LOG(THETA(17))
MU_13 = LOG(THETA(18))

MTT_TNF = EXP(MU_6 + ETA(6))
```

```
S0_TNF = EXP(MU_7 + ETA(7))
EMAX_TNF = EXP(MU_8 + ETA(8))
EC0_TNF = EXP(MU_9 + ETA(9))
ECMTT_TNF = EXP(MU_10 + ETA(10))
TMAX_TNF = EXP(MU_11 + ETA(11))
EGAM_TNF = EXP(MU_12 + ETA(12))
TC50_TNF = EXP(MU_13 + ETA(13))

KOUT_TNF = 1 / MTT_TNF
KIN_TNF = S0_TNF * KOUT_TNF
ECKOUT_TNF = 3 / ECMTT_TNF
ECKIN_TNF = EC0_TNF * ECKOUT_TNF

; --- IL6
MU_14 = LOG(THETA(19))

TVS0_IL6 = THETA(20)
IF (STUDY == 3 .OR. STUDY == 4 .OR. STUDY == 6) TVS0_IL6 = THETA(21)
MU_15 = LOG(TVS0_IL6)

MU_16 = LOG(THETA(22))
MU_17 = LOG(THETA(23))

MTT_IL6 = EXP(MU_14 + ETA(14))
S0_IL6 = EXP(MU_15 + ETA(15))
D1SLP_IL6 = EXP(MU_16 + ETA(16))
D2SLP_IL6 = EXP(MU_17 + ETA(17))

KOUT_IL6 = 5 / MTT_IL6
KIN_IL6 = S0_IL6 * KOUT_IL6

A_0(1) = SOIL
A_0(2) = 0
A_0(3) = S0_TNF
A_0(4) = EC0_TNF
A_0(5) = EC0_TNF
A_0(6) = EC0_TNF
A_0(7) = S0_IL6
A_0(8) = S0_IL6
A_0(9) = S0_IL6
A_0(10)= S0_IL6
A_0(11)= S0_IL6

$DES
CP = A(2) / V1
EC50 = A(6)
TOL_TNF = TMAX_TNF * CP / (TC50_TNF + CP)
EFF_TNF = EMAX_TNF * CP**EGAM_TNF / (EC50**EGAM_TNF + CP**EGAM_TNF)

CTNF = A(3)
EFF1_IL6 = D1SLP_IL6 * (CTNF / S0_TNF - 1)
EFF2_IL6 = D2SLP_IL6 * CP

DADT(1) = -VMAX * (A(1) / V1) / (KM + (A(1) / V1))
DADT(2) = -VMAX * (A(2) / V1) / (KM + (A(2) / V1))
DADT(3) = KIN_TNF * (1 + EFF_TNF) -KOUT_TNF * A(3)
DADT(4) = ECKIN_TNF * (1 + TOL_TNF) -ECKOUT_TNF * A(4)
DADT(5) = ECKOUT_TNF * A(4) -ECKOUT_TNF * A(5)
DADT(6) = ECKOUT_TNF * A(5) -ECKOUT_TNF * A(6)
DADT(7) = KIN_IL6 * (1 + EFF1_IL6 + EFF2_IL6) -KOUT_IL6 * A(7)
DADT(8) = KOUT_IL6 * A(7)          -KOUT_IL6 * A(8)
DADT(9) = KOUT_IL6 * A(8)          -KOUT_IL6 * A(9)
DADT(10)= KOUT_IL6 * A(11)         -KOUT_IL6 * A(10)
```

```
DADT(11)= KOUT_IL6 * A(9)         -KOUT_IL6 * A(11)

$ERROR
NIPRD = EBASE + A(1) / V1        ; BASE+ETX (EU/L)
IF (DVID == 1) NIPRD = A(3)     ; TNFA (ng/L)
IF (DVID == 2) NIPRD = A(10)    ; IL6 (ng/L)
IPRED = LOG(NIPRD)

IRES  = DV - IPRED
W     = SQRT(SIGMA(1,1))
IF (DVID == 1) W = SQRT(SIGMA(2,2))
IF (DVID == 2) W = SQRT(SIGMA(3,3))
IWRES = IRES / W

Y     = IPRED + EPS(1)
IF (DVID == 2) Y = IPRED + EPS(3)

LOQ = LOG(LLOQ)

IF (TYPE.EQ.0 .AND. DVID.EQ.1) THEN
F_FLAG = 0
Y = IPRED + EPS(2)
ENDIF

IF (TYPE.EQ.1 .AND. DVID.EQ.1) THEN
DUM = (LOQ - IPRED) / W
CUMD = PHI(DUM)
F_FLAG = 1
Y = CUMD
MDVRES = 1
ENDIF

IF (TYPE.EQ.0 .AND. DVID.EQ.2) THEN
F_FLAG = 0
Y = IPRED + EPS(3)
ENDIF

IF (TYPE.EQ.1 .AND. DVID.EQ.2) THEN
DUM = (LOQ - IPRED) / W
CUMD = PHI(DUM)
F_FLAG = 1
Y = CUMD
MDVRES = 1
ENDIF

$THETA  (1E-6, 442143)        ; 1 VMAX; EU/h
$THETA  (1E-6, 12325.3)       ; 2 KM; EU/L
$THETA  (1E-6, 34.7795)       ; 3 V1; L
$THETA  (1E-6, 157.145)       ; 4 BASE; EU/L
$THETA  (1E-6, 1807.02)       ; 5 BASE6; EU/L
$THETA  (1E-6, 132.529)       ; 6 SCALE; -
$THETA  (0, 0.399308, 1) FIX  ; 7 MIX; -
$THETA  (1E-6, 1.05168)       ; 8 MTT; h
$THETA  (1E-6, 280.522)       ; 9 S0; ng/L
$THETA  (1E-6, 3.00483)       ; 10 S0; ng/L
$THETA  (1E-6, 66.4359)       ; 11 S0; ng/L
$THETA  (1E-6, 25.2023)       ; 12 S0; ng/L
$THETA  (1E-6, 2552.66)       ; 13 EMAX; -
$THETA  (1E-6, 296.491)       ; 14 EC0; EU/L
$THETA  (1E-6, 6.40055)       ; 15 ECMTT; h
$THETA  (1E-6, 43708.4)       ; 16 TMAX; -
$THETA  (1E-6, 2.11093)       ; 17 EGAM; -
$THETA  (1E-6, 29802.7)       ; 18 TC50; EU/L
```

```
$THETA  (1E-6, 1.42911)       ; 19 MTT; h
$THETA  (1E-6, 49.3472)       ; 20 S0; ng/L
$THETA  (1E-6, 8.03618)       ; 21 S0; ng/L
$THETA  (1E-6, 0.968638)      ; 22 D1SLP; -
$THETA  (1E-6, 0.00955112)    ; 23 D2SLP; -

$OMEGA  0.000625  FIX         ; 1 VMAX ETX
$OMEGA  BLOCK(2)
 1.44404                      ; 2 KM ETX
 -0.383922 0.564858           ; 3 V1 ETX
$OMEGA  0.916907              ; 4 BASE ETX
$OMEGA  0.000625  FIX         ; 5 PROP ETX
$OMEGA  0.662022              ; 6 MTT TNF
$OMEGA  0.294453              ; 7 S0 TNF
$OMEGA  0.000625  FIX         ; 8 EMAX TNF
$OMEGA  0.000625  FIX         ; 9 EC50 TNF
$OMEGA  0.000625  FIX         ; 10 TMTT TNF
$OMEGA  0.000625  FIX         ; 11 TMAX TNF
$OMEGA  0.000625  FIX         ; 12 EGAM TNF
$OMEGA  0.000625  FIX         ; 13 TC50 TNF
$OMEGA  0.229546              ; 14 MTT IL6
$OMEGA  0.638651              ; 15 S0 IL6
$OMEGA  0.000625  FIX         ; 16 D1SLP IL6
$OMEGA  0.000625  FIX         ; 17 D2SLP IL6

$SIGMA  0.105128              ; 1 PropRES ETX
$SIGMA  0.235298              ; 2 PropRES TNFA
$SIGMA  0.254526              ; 3 PropRES IL6

$PHIS FILE=../../run045.phi
$ESTIMATION METHOD=IMP LAPLACIAN INTERACTION MAPITER=1 MAPINTER=1 NITER=300
            AUTO=1 GRD=SN(2-3) RANMETHOD=3SP PRINT=1 POSTHOC NOABORT
MSFO=run045_msfi
$COVARIANCE PRINT=E PRINT=R UNCONDITIONAL

$TABLE ID SID STUDY DVID DGRP TIME CMT MDV OMIT CWRES IWRES IPRED ONEHEADER
APPEND NOPRINT FILE=sdtab046
$TABLE ID MTT_IL6 S0_IL6 D1SLP_IL6 D2SLP_IL6 ETAS(1:LAST) ONEHEADER NOAPPEND
NOPRINT FILE=patab046
;$TABLE ID WT ABSD ONEHEADER NOAPPEND NOPRINT FILE=cotab001
;$TABLE ID STUDY INFL FLUID ONEHEADER NOAPPEND NOPRINT FILE=catab001
```
